# Monitoring and Combating Waterpipe Tobacco Smoking Through Surveillance and Taxation

**DOI:** 10.2196/40177

**Published:** 2023-03-23

**Authors:** Randa K Saad, Adna Maiteh, Rima Nakkash, Ramzi G Salloum, Ali Chalak, Niveen M E Abu-Rmeileh, Yousef Khader, Mohannad Al Nsour

**Affiliations:** 1 Global Health Development | Eastern Mediterranean Public Health Network Amman Jordan; 2 Department of Health Promotion and Community Health Faculty of Health Sciences American University of Beirut Beirut Lebanon; 3 Department of Global and Community Health College of Health and Human Services George Mason University Fairfax, VA United States; 4 Department of Health Outcomes and Biomedical Informatics College of Medicine University of Florida Florida, FL United States; 5 Department of Agriculture Faculty of Agricultural and Food Sciences American University of Beirut Beirut Lebanon; 6 Institute of Community and Public Health Birzeit University Birzeit Occupied Palestinian Territory; 7 Public Health Faculty of Medicine Jordan University of Science and Technology Irbid Jordan

**Keywords:** waterpipe tobacco, smoking, tobacco taxation, Global Tobacco Surveillance System, GTSS, Eastern Mediterranean Region, tobacco, public health, surveillance, taxation

## Abstract

Waterpipe tobacco smoking (WTS) is a traditional tobacco use method that originated in the Eastern Mediterranean Region (EMR) and has resurged in recent decades. WTS rates in the EMR are the highest worldwide, especially among youth, exceeding cigarette-smoking rates in select jurisdictions. Despite its documented harm, the growing prevalence of WTS has been met with a poor regulatory response globally. At the epicenter of the WTS epidemic, countries in the EMR are in urgent need of effective tobacco control strategies that consider the particularities of WTS. A roundtable session, titled “Monitoring and Combating WTS Through Taxation and the Global Tobacco Surveillance System (GTSS),” was held as part of the 7th Eastern Mediterranean Public Health Network’s regional conference. The session provided an overview of evidence to date about WTS policy control, the taxation of WTS, volumetric choice experiments for tobacco control research, and monitoring WTS patterns and control policies among adults and youth through the GTSS. The session highlighted the need to update the regulation of WTS in the current global tobacco control policy frameworks and the need for developing tailored, evidence-based, and WTS-specific regulations to complement current tobacco control policy frameworks. Raising taxes to increase the price of tobacco products is the single most effective tobacco control measure, and these taxes can fund expanded government health programs. The effectiveness of taxation can be measured via volumetric choice experiments, which allow for the estimation of a complete set of own-price and cross-price elasticities that are instrumental for fiscal policy simulations. Finally, the surveillance of WTS (for example, through the GTSS) is critical to informing policy and decision makers. The Global Youth Tobacco Survey (GYTS) and Global Adult Tobacco Survey (GATS) are 2 GTSS products that provide nationally representative data among students aged 13-15 years and persons ≥15 years, respectively.

## Introduction

The Eastern Mediterranean Region (EMR) has the lowest average prices of tobacco products among all World Health Organization (WHO) regions [1] and is the only region in which smoking prevalence has been projected to increase by 2025 [2]. However, there is little research on the economics of tobacco, and the majority of demand elasticities estimates for tobacco products are for cigarettes [3]. In a recent report on the performance of cigarette tax policies, the EMR as a whole had the second lowest score [4].

Waterpipe tobacco smoking (WTS) is a traditional tobacco use method that originated in the EMR [5], and its use continues to increase globally, especially among youth and young adults [6-8], exceeding cigarette-smoking rates [9]. Despite its documented harm, including its link with lung cancer [9], respiratory illnesses, periodontal diseases, and low birth weight [10], the growing prevalence of WTS has been met with a poor regulatory response globally [9]. At the epicenter of the WTS epidemic, countries in the EMR need effective tobacco control strategies that consider the specific particularities of WTS.

Article 6 of the WHO Framework Convention on Tobacco Control (FCTC) supports taxation, and where appropriate, pricing policies, to curb the use of tobacco products [11]. Additionally, the MPOWER policy package of effective tobacco control policies stresses that raising the price of tobacco products through taxation is the most effective way to reduce smoking [12]. However, evidence to date has been largely limited to cigarettes, and thus, evidence to support fiscal measures to curb WTS is scarce. This evidence gap was acknowledged in a 2015 WHO advisory note and more recently in a National Cancer Institute monograph on the economics of tobacco control [13]. A limited number of published studies exist on the economics of tobacco control in the EMR. For the most part, these studies have generally focused on examining cigarette smoking. Given that WTS prevalence exceeds that of cigarette smoking among certain populations in the region, there is a need for models that more accurately capture WTS particularities. For example, the WHO recommends considering scenarios where taxation is on the individual user (at the consumer level) and possibly taxing the waterpipe parts and accessories [14].

In addition to the importance of raising taxes on tobacco products, the MPOWER policy package stresses the importance of monitoring tobacco use, as it is a cross-cutting activity and involves periodically collecting nationally representative population-based youth and adult data on key indicators of tobacco use [12]. To that end, and to assist countries in establishing tobacco surveillance and control programs, the WHO, US Centers for Disease Control and Prevention (CDC), and Canadian Public Health Association initiated the Global Tobacco Surveillance System (GTSS) [15]. The GTSS aims to build the capacity of countries to plan, implement, monitor, and evaluate tobacco control interventions, providing central targets and indicators for the WHO FCTC and the WHO MPOWER technical package, including exposure to mass media campaigns and cost-related indicators [15]. Currently, the GTSS collects data through the Global Youth Tobacco Survey (GYTS), the Global School Personnel Survey, the Tobacco Questions for Surveys (TQS), and the Tobacco Questions for Surveys of Youth (TQS-Youth) [15]. With the Global Adult Tobacco Survey (GATS) being the nationally representative household survey among individuals 15 years of age or older [16], GTSS data can be used to inform policy makers about the tobacco problem in their country, leading to new policy decisions on tobacco prevention and control.

This viewpoint aims to report on the learnings of a roundtable session that focused on the various evidence-based WTS policies; describe the methods used to assess the price elasticity of demand for WTS and the cross-price elasticity between cigarettes and WTS in Lebanon, Jordan, Palestine, and Egypt; and explain how the GTSS can provide important data for the EMR countries on WTS use and related WHO MPOWER policy measures, including exposure to mass media campaigns and cost-related indicators.

## Roundtable Description

A roundtable session was held on November 17, 2021, as part of the 7th Eastern Mediterranean Public Health Network’s regional conference to discuss the most recent evidence on WTS in the EMR, including monitoring and controlling demand through taxation. The roundtable included oral presentations and an interactive discussion of questions and comments from participants. The following topics were presented to address the roundtable objectives: WTS policy control, taxation of WTS, volumetric choice experiments (VCEs) for tobacco control research, and monitoring WTS and control policies among adults and youth through the GTSS.

## Waterpipe Tobacco Control Policy: Evidence to Date

Mainly driven by youth uptake, WTS has increased over the past 2 decades in many countries around the world, including in the EMR [17]. Factors that may have contributed to the increased use and prevalence of WTS include the introduction of flavoring with reduced harshness, the misperception of it being healthier than other tobacco products, its affordability, the quick lit charcoal, the allure of WTS on social media, the social acceptance of waterpipe cafes, the lack of waterpipe-specific policies and regulations, and the immigration patterns from countries with a high prevalence of use to low-prevalence countries [17]. Some unique features of WTS include the fact that it is a stationary and time-consuming tobacco use method, with the usual duration of each session lasting 30 minutes or more [18-20], often used in dedicated cafes and restaurants; flavor is the main product focus; and sharing it with others is a dominant product feature. WTS involves several accessories, including charcoal, the hose, and the device itself, and is self-assembled.

A systematic review of interventions for WTS concluded that there is a lack of evidence of the effectiveness for most waterpipe control interventions, with few showing promising results, and recommended that higher-quality interventions are needed [21].

The FCTC is an international health treaty adopted by the World Health Assembly in 2003. The WHO FCTC is largely based on evidence collected from cigarette policy effectiveness [22]. Although this framework may help in the regulation of other tobacco products, the global rise in their prevalence, particularly WTS [6], has introduced a number of issues [23]. The expansion of producers, exporters, and manufacturers of waterpipe tobacco and accessories poses a significant challenge to WTS legislation. This is in stark contrast to the tobacco industry, which is dominated by a few global corporations [24]. WTS also has distinct characteristics from cigarette smoking, such as the use of charcoal briquettes, a large apparatus and hose available in a variety of sizes, and a diverse range of tobacco flavors and packaging modes, all of which may necessitate an alternative regulatory mechanism to supplement the current WHO FCTC–recommended framework [25].

Existing regulations must be adapted to effectively address WTS, in addition to the need for waterpipe-specific legislation to meet these unique difficulties. For example, current tobacco control policy frameworks in many jurisdictions do not define whether health warning labels should be applied to waterpipe apparatuses and other accessories, and if they do, there is no guidance on how to use them in practice [26]. This is essential because persons who smoke at waterpipe-serving establishments are rarely exposed to waterpipe tobacco packages; instead, they are only offered a waterpipe apparatus [26]. Despite this, the health warning labels on present waterpipe tobacco packets include a number of deceptive characteristics [27], including incorrect ingredients labeling [28], and do not follow the WHO FCTC’s guidelines [29]. The introduction of dangerous waterpipe tobacco alternatives (called “herbal” or “nontobacco”), which may be excluded from tobacco control legislation but are sold and consumed alongside waterpipe tobacco and may be indistinguishable from it, further complicates matters [30].

There are regulatory gaps in place to regulate WTS across the world, and these gaps could jeopardize existing tobacco control strategies. There is a chance to assess current and prospective policy alternatives for reducing WTS and use that information to establish a new tobacco control policy framework. Tobacco control researchers play a vital role in providing policy makers with the necessary evidence to propose effective legislation. The WHO FCTC secretariat should consider establishing a scientific working group to explore WTS regulatory concerns and propose a complementary framework to the WHO FCTC. Countries that bear the brunt of the load of tobacco-related diseases and have experience in regulating WTS should be involved [31].

When it comes to reducing the uptake of smoking among young people, tobacco product costs are an important aspect to consider because decreasing affordability is the most effective strategy [32]. To minimize tobacco consumption, the WHO FCTC proposes that taxation policies consider the price elasticity of demand, and that all tobacco products be taxed equally to avoid unexpected consequences such as product substitution [2].

## Taxation of WTS

There are direct costs associated with tobacco use, including health care cost to treat tobacco-related diseases, and non–health care costs, such as transportation to a clinic and the time of family members providing care [33]. There are also indirect costs of tobacco use, associated with the reduction in potential economic productivity due to morbidity and premature mortality [33,34]. Additionally, there are 3 types of societal costs: external, internal, and indirect. External societal costs are the costs that tobacco users impose on others such as through secondhand smoking. Internal societal costs result from the information failures in the market that can be thought of as external costs. Indirect societal costs are costs paid by tobacco users and their families, incurred as a result of tobacco (eg, out-of-pocket costs for health care to treat diseases caused by smoking) [33]. The latest estimates of the total worldwide economic cost of tobacco smoking are above US $1.4 trillion (2012), which is 1.8% of the world’s annual GDP [34]. Direct health care costs alone are estimated at US $443 billion dollars, whereas indirect costs, incurred due to the loss of productivity as a result of morbidity and mortality, are estimated at US $357 billion and US $657 billion, respectively [34].

Taxation is an evidence-based tool for tobacco control. First, taxation promotes public health. Depending on the tax structure, taxation can have a higher impact on demand among vulnerable individuals such as the young population, whose demand is more elastic than the demand of older adults. This is also true for less educated and low-income individuals who are disproportionately affected by the burden of tobacco smoking. Second, taxation is an efficient revenue generation strategy for governments as smoking is relatively inelastic, where consumers have a lower behavioral response when the price is raised compared to products with higher elasticity [35]. Third, from a societal perspective, taxation corrects for the external costs of tobacco borne by members of society other than the smoker.

There is a substantial body of research over many decades and from many countries, showing that significantly increasing the excise tax and price of tobacco products to reduce their affordability is the single most consistently effective tool for reducing tobacco use [13]. There are a variety of tobacco taxes used: some are based on sales taxes or value-added taxes; some are in the form of customs duties on tobacco leaf and product imports or exports; and some are implicit taxes when governments monopolize production and distribution. There are also excise taxes, which are of the most interest given their specificity to the tobacco products. Excise taxes are of 2 types. The first type is the specific tax, which is based on the weight or volume of the tobacco used. The benefit of this type of tax is that it reduces price gaps, deters tax avoidance, is easy to administer, and stabilizes tax revenue. The second type of excise tax is ad valorem, which is based on the commercial value of the product. The benefits of the ad valorem tax are that it adjusts with inflation and is progressive, imposing higher taxes on higher-priced products (ie, taxes higher-income individuals). However, it leads to greater price gaps and incentivizes tax avoidance.

Early efforts to examine the price elasticity of demand for noncigarette products have provided sufficient evidence of the effectiveness of tax and price increase in decreasing demand. A systematic review concluded that there is positive substitution between cigarette and noncigarette products, suggesting that tax and price increase should be simultaneous and comparable across all tobacco products [36].

The Eastern Mediterranean Consortium on the Economics of WTS is an ongoing project examining the economics of WTS. This is a collaboration between the American University of Beirut, Jordan University of Science and Technology, Birzeit University, and Ain Shams University and is funded by Cancer Research UK and the International Development Research Centre (IDRC). In its most recent study, the Consortium examined the price elasticities of waterpipe tobacco products and cigarette products [3]. The WHO FCTC recommends that the price elasticity of demand be taken into account in taxation policy to reduce tobacco use [2]. The price elasticity of a product measures how sensitive the demand for it responds to a change in its own price (own-price elasticity) or in the price of other related products (cross-price elasticity) [37]. Overall, the price elasticity observed for WTS and cigarette smoking varied by product and across countries, with the demand for premium cigarettes being price elastic (range −1.0 to −1.2) and that for premium waterpipe tobacco being highly elastic in Lebanon (−1.9), moderately elastic in Jordan (−0.6), and inelastic in Palestine (0.2) [3].

## VCEs for Tobacco Control Research

The methodology that was used to guide the research of the Eastern Mediterranean Consortium on the Economics of WTS includes VCEs for tobacco control research. All economic problems are rooted in the issue of choices that need to be made as resources are scare (opportunity cost). Choices reveal information about preferences of consumers, which is instrumental for understanding the costs and benefits attendant to health policies and interventions. This informs how responsive consumers would be reacting to changes in prices. The most obvious avenue to observe choices, and thus preferences, is to observe market choices, using the revealed preference methods. The methods involve collecting market data and inferring measures of demand and preference intensities mainly through the vehicle of elasticities. The advantage of these methods is that they have a high face validity as they rely on real-life data [38]. However, in some situations, it is limited by the lack of availability of products, that is, it cannot measure choice preference for products that do not exist on the market. Therefore, economists sometimes rely on observing hypothetical choices by means of stated preference (SP) methods. These methods present consumers with hypothetical products and collect what they state they will buy at experimentally varied prices [39]. However, these methods lack consequentiality, often resulting in respondents inflating their willingness to purchase and pay for the products under study (hypothetical bias) [39].

The most common SP approach used in marketing and economic research is discrete choice experiments (DCEs), which are an attribute-based, hypothetical, and survey-based tool for measuring preference and value [40,41]. DCEs incorporate the notion of opportunity cost and choice. DCEs can be disadvantageous in the demand analysis of certain products such as WTS. Therefore, VCEs were developed as an alternative to the SP methods. DCEs force respondents to choose one and only one option, which is often restrictive compared to real market situations. VCEs, in contrast, accommodate simultaneous choices of multiple options and accommodate “quantitative” or “volumetric” choices (ie, the number of units selected). It does this by simultaneously offering multiple distinct volumetric choices across competing products. Data from Jordan, Palestine, and Lebanon show the elasticity of demand for waterpipe tobacco products and the existence of substitution between tobacco products, which should be considered when formulating tobacco taxation strategies [42]. Thus, VCEs allow for the estimation of a complete set of own-price and cross-price elasticities that are instrumental for the purpose of fiscal policy simulations [3]. This technique is useful in forecasting how consumers will respond to policies that increase the prices of products such as tobacco and by how much such policies may increase revenue to governments. This is illustrated in a study where the elasticity estimates from the VCE were fed to a simulation model that was adapted to forecast the effects of taxation policies. Specifically, the model used country-specific and market share–specific price and consumption data from the WHO and United Nations (UN) Comtrade, in addition to the VCE’s elasticity estimates obtained from nationally representative surveys conducted in Lebanon, Jordan, and Palestine. It then forecasted the effects of specific excise taxes, which met a 35.9% tax burden on waterpipe tobacco in Lebanon and Jordan (in line with the global average) and doubled government revenues from excise duties in Palestine [43].

## Monitoring WTS and Control Policies Among Adults and Youth Through the GTSS

As COVID-19 spread in the EMR, there was a renewed focus on curbing smoking as 17 countries/sites temporarily banned WTS in indoor and outdoor public places during the pandemic [44]. Additionally, the CDC, in collaboration with the Eastern Mediterranean Public Health Network and Vital Strategies, launched the “United Against Tobacco and COVID” campaign in Jordan, Egypt, Iraq, and Palestine. This project aimed to warn on the harms of tobacco, in accordance with the MPOWER policy package recommendations [12]. The project intended to raise awareness of the harms of smoking, especially during the COVID-19 pandemic, through media coverage. This project used evidence-based information and data to develop country-tailored and culturally appropriate messages to disseminate, educate, and promote smoking cessation to reduce COVID-19 morbidity and mortality.

This campaign, similar to all antitobacco interventions, programs, and policies, relied on accurate measures of tobacco use to determine the target audience as well as the target messages that need to be addressed within each county. Indeed, to plan any tobacco control strategy or intervention effectively, counties need accurate data to inform implementation and increase the likelihood of success.

One approach of obtaining such data is through globally standardized surveys such as the GTSS, which systematically monitors youth and adult tobacco use as well as key tobacco control indicators (the WHO FCTC and MPOWER) [15]. It also monitors and enhances the capacity to design, implement, and evaluate tobacco control policies. The GYTS, a nationally representative school-based survey of students aged 13-15 years, is the largest public health surveillance system ever developed and maintained [22]. The survey is self-administered in the classroom setting using a paper-and-pencil questionnaire [22]. It contains a core questionnaire with optional modules for countries to add additional questions. Between 1999-2020, the GYTS was implemented in 188 countries/sites [45]. This standard, systematic, and consistent process generates comparable data within and across countries. The GYTS *Shisha (or Waterpipe)* module is an optional module that can be used [46].

These collected surveillance data are helpful in designing, guiding, monitoring, and evaluating tobacco control interventions, programs, and policies at the country level. One key goal for such data is to highlight the extent of the tobacco epidemic in countries, including WTS, and thus generate interest and support for tobacco control policy, and those for WTS, among key policy makers and stakeholders. For example, if policy makers are considering a smoke-free ban, data will serve to educate stakeholders about the extent of exposure to secondhand smoke within the country and thus support the ban.

Additionally, using surveillance data to understand the tobacco market within a country is one of the strategies to ensure that tobacco taxation, including taxation on WTS, is effective. Surveys enable the identification of changes in consumption patterns so that existing policies can be adjusted accordingly. Authorities might ask for more information on certain aspects such as consumption patterns and attitudes toward WTS among youth. This information is crucial to assess the tobacco industry’s marketing power and can inform discussions on elasticities.

In the EMR, GYTS WTS data are available from 20 countries/sites from 2005 to 2016. Table 1 provides the prevalence rates of current WTS among students aged 13-15 years in these 20 countries/sites. The overall prevalence rates ranged from 34.8% in Lebanon (2011) to 3.8% in Morocco (2016). The prevalence rates were significantly higher among boys than girls in all but 2 countries/sites.

The GATS is a nationally representative household-based face-to-face interview survey of individuals aged 15 years or older, using a global standard protocol and electronic data collection methodology [16]. It similarly includes a core questionnaire with optional questions and options for countries to add additional questions [16]. Since 2008, the GATS has been implemented in 36 countries representing more than 70% of the world’s adult population [22]. It contains WTS core questions including *Current WTS* and *Frequency of WTS*, as well as an optional WTS module [16].

In the EMR, GATS WTS data are available from 4 countries from 2009 to 2019. Table 2 provides the prevalence rates of current WTS among individuals aged 15 years and older in these 4 countries. The prevalence rates were as follows: 6.7% in Saudi Arabia in 2019 (9.7% males and 2.3% females); 3.4% in Qatar in 2013 (4.9% males and 1.6% females); 3.3% in Egypt in 2009 (6.2% males and 0.3% females); and 3.0% in Pakistan in 2014 (4.7% males and 1.1% females).

Unfortunately, monitoring rates through standardized national population-based surveillance surveys vary by country income group, with better coverage achieved in higher-income countries. As per the WHO’s most recent global report on trends in the prevalence of tobacco use from 2000-2025, the lowest population coverage by such surveys is in the EMR, with only 88% of the population living in 76% of the EMR having sufficient available data to calculate tobacco use trends [2].

To that end, the TQS is beneficial. The TQS includes a subset of 22 key questions from the GATS for adults [47], and the TQS-Youth contains a subset of 21 key questions from the GYTS for youth [48]. These questions are integrated into ongoing surveys (health or otherwise, such as the WHO Stepwise Approach to Surveillance surveys) for sustainable monitoring and global consistency. Between 2008 and 2021, 100 countries completed surveys with TQS integration.

However, in addition to the data that are collected by the standardized surveillance surveys, policy makers may require data about the composition of tobacco and WTS products, including flavors and their production and marketing costs by brand. This additional information can provide a better understanding of the market power, market share, and behavior of the tobacco industry within a country. It can also inform discussions on elasticities, including how consumers choose specific brands or flavors.

**Table 1:** Prevalence of current waterpipe smoking among students 13-15 years old, by sex—Global Youth Tobacco Survey, Eastern Mediterranean Region countries/sites, 2005-2016.

Country/site (year)	Current waterpipe smoking				
	Overall (%)	Boys (%)	Girls (%)	*P* value (boys vs girls)	
Lebanon (2011)	34.8	39.3	31.0	.03	
Jordan (2014)	26.7	34.5	18.4	.001	
Syria (2010)	19.2	24.7	13.8	.04	
Bahrain (2015)	18.3	26.1	10.2	.001	
Palestine—West Bank (2016)	17.9	23.4	12.4	.001	
Qatar (2013)	17.1	24.6	10.1	.001	
Palestine—Gaza Strip (2013)	16.2	22.0	11.1	.01	
Yemen (2014)	14.1	17.1	9.3	.001	
Kuwait (2016)	13.9	19.5	9.0	.001	
United Arab Emirates (2005)	12.4	16.3	7.6	.001	
Djibouti (2013)	11.6	10.2	12.8	.20	
Iran (2016)	11.2	14.3	8.5	.03	
Iraq (2014)	10.6	15.1	5.3	.01	
Sudan (2014)	9.9	13.0	5.4	.01	
Saudi Arabia (2010)	9.5	13.3	6.1	.04	
Oman (2016)	7.4	11.6	3.7	.03	
Egypt (2014)	5.9	7.2	4.1	.09	
Tunisia (2010)	5.8	10.1	2.1	.001	
Libya (2010)	4.3	6.0	2.6	.001	
Morocco (2016)	3.8	5.0	2.6	.04	

**Table 2:** Prevalence of current waterpipe smoking among individuals aged 15+ years, by sex—Global Adult Tobacco Survey, Eastern Mediterranean Region countries, 2009-2019.

Country (year)	Current waterpipe smoking				
	Overall (%)	Male (%)	Female (%)	*P* value^a^ (males vs females)	
Saudi Arabia (2019)	6.7	9.7	2.3	.001	
Qatar (2013)	3.4	4.9	1.6	.001	
Egypt (2009)	3.3	6.2	0.3	.001	
Pakistan (2014)	3.0	4.7	1.1	.001	

^a^Significance at *P*<.05

## Conclusion

This paper provides an overview of WTS regulatory and surveillance issues in the EMR and discusses examples of success in taxation at the level of the consumer as well as the methods used to assess the feasibility and effectiveness of taxation on demand. The immense need to collect data on WTS using the GTSS was also stressed, to enable policy makers to make better informed decisions.

## Recommendations and Key Areas for Improvement

- There is a need to update the regulation of WTS in the current global tobacco control policy frameworks.
- There is a need to develop tailored and evaluated WTS-specific regulatory frameworks to complement current tobacco control policy frameworks.
- Raising taxes to increase the price of tobacco products is the single most effective tobacco control measure, and this also applies to WTS.
- Increased taxes from WTS can fund expanded government health programs.
- VCEs allow for the estimation of a complete set of own-price elasticities and cross-price elasticities for WTS products, which is instrumental for the purpose of fiscal policy simulations.
- Gaining political buy-in is key to adopting key tax reforms relating to tobacco and WTS, and to this end, data from standardized validated surveillance systems are crucial.
- Data collection on WTS through the GTSS is critical to inform and sensitize policy makers and decision makers from the EMR about the public health and socioeconomic burdens caused by WTS, as well as the growing use among females and youth.
- The GYTS and GATS can provide nationally representative data among students aged 13-15 years and ≥15 years, respectively, on WTS, and countries can include an optional waterpipe module.
- As a cost-effective measure, and to ensure some data on WTS are being collected, it is suggested to include the TQS on WTS within other surveys that countries are already implementing.
- Data on WTS that are generated from the GYTS and GATS are the most valuable when disseminated in a way that will gain the attention of key stakeholders, especially policy makers and the media, as they are the most important audience if these data are to cause a ripple effect. Effective data dissemination is crucial, and so is engaging partners to help translate important information to key decision makers, who have the authority to change tobacco control policy.

## Abbreviations

CDC: Centers for Disease Control and Prevention
DCE: discrete choice experiment
EMR: Eastern Mediterranean Region
FCTC: Framework Convention on Tobacco Control
GATS: Global Adult Tobacco Survey
GTSS: Global Tobacco Surveillance System
GYTS: Global Youth Tobacco Survey
IDRC: International Development Research Centre
SP: stated preference
TQS: Tobacco Questions for Surveys
TQS-Youth: Tobacco Questions for Surveys of Youth
UN: United Nations
VCE: volumetric choice experiment
WHO: World Health Organization
WTS: waterpipe tobacco smoking

## Data Availability

Data sharing not applicable to this paper as no data sets were generated or analyzed during the current study.
